# Evaluation of the Antimicrobial Activity of Different Antibiotics Enhanced with Silver-Doped Hydroxyapatite Thin Films

**DOI:** 10.3390/ma9090778

**Published:** 2016-09-16

**Authors:** Daniela Predoi, Cristina Liana Popa, Patrick Chapon, Andreea Groza, Simona Liliana Iconaru

**Affiliations:** 1 National Institute of Materials Physics, Atomistilor Street, No. 405A, P.O. Box MG 07, 077125 Magurele, Romania; cristina.lyana@gmail.com (C.L.P.); simonaiconaru@gmail.com (S.L.I.); 2 Horiba Jobin Yvon S.A.S., 16-18, rue du Canal, 91165 Longjumeau Cedex, France; patrick.chapon@horiba.com; 3 National Institute for Laser, Plasma and Radiation Physics, 409 Atomistilor Street, P.O. Box MG 36, 077125 Magurele, Romania; andreea@infim.ro

**Keywords:** silver, hydroxyapatite, thin films, antibiotics, antibacterial activities

## Abstract

The inhibitory and antimicrobial effects of silver particles have been known since ancient times. In the last few years, a major health problem has arisen due to pathogenic bacteria resistance to antimicrobial agents. The antibacterial activities of new materials including hydroxyapatite (HAp), silver-doped hydroxyapatite (Ag:HAp) and various types of antibiotics such as tetracycline (T-HAp and T-Ag:HAp) or ciprofloxacin (C-HAp and C-Ag:HAp) have not been studied so far. In this study we reported, for the first time, the preparation and characterization of various thin films based on hydroxyapatite and silver-doped hydroxyapatite combined with tetracycline or ciprofloxacin. The structural and chemical characterization of hydroxyapatite and silver-doped hydroxyapatite thin films has been evaluated by X-ray diffraction (XRD) and Fourier transform infrared spectroscopy (FTIR). The morphological studies of the HAp, Ag:HAp, T-HAp, T-Ag:HAp, C-HAp and C-Ag:HAp thin solid films were performed using scanning electron microscopy (SEM). In order to study the chemical composition of the coatings, energy dispersive X-ray analysis (EDX) and glow discharge optical emission spectroscopy (GDOES) measurements have been used, obtaining information on the distribution of the elements throughout the film. These studies have confirmed the purity of the prepared hydroxyapatite and silver-doped hydroxyapatite thin films obtained from composite targets containing Ca_10−*x*_Ag*_x_*(PO_4_)_6_(OH)_2_ with *x*_Ag_ = 0 (HAp) and *x*_Ag_ = 0.2 (Ag:HAp). On the other hand, the major aim of this study was the evaluation of the antibacterial activities of ciprofloxacin and tetracycline in the presence of HAp and Ag:HAp thin layers against *Staphylococcus aureus* and *Escherichia coli* strains. The antibacterial activities of ciprofloxacin and tetracycline against *Staphylococcus aureus* and *Escherichia coli* test strains increased in the presence of HAp and Ag:HAp thin layers.

## 1. Introduction

With the development of bioengineering, new and improved biomaterials have been created and perfected in order to be used in different biomedical applications [1,2]. One of the most promising biomaterials is hydroxyapatite (HAp). As a member of the calcium phosphate ceramics, hydroxyapatite (Ca_10_(PO_4_)_6_OH_2_) is a major constituent of human bones and teeth [3,4]. Due to its similarity to natural hard tissue, synthetic hydroxyapatite has been already widely used for various applications such as implant material or coating material for metallic implants [5,6]. Recent studies have shown that HAp has remarkable osteoconductivity and osteoinductivity, being able to form bonds with natural human hard tissue [7,8,9,10]. Clinically, HAp has been used as a filling material for obstructing tooth cavities or bone fractures [2]. However, since synthetic hydroxyapatite has very low mechanical strength, it is usually used as a coating material for various metallic implants, facilitating osteointegration and thus increasing the success rate of orthopedic implants [11,12,13,14,15,16]. Considering its good biocompatibility, hydroxyapatite is a good candidate for medical applications. Recently, researchers have discovered that different organic substances such as proteins or amino acids adsorbed on its surface transform it into an ideal environment for bacterial replication [5]. These bacteria give rise to post-surgical infection and may determine the formation of a biofilm [17]. The microorganisms which cause the biofilm are usually resistant to antimicrobial agents [17]. In many cases, a consequence of post-operatory infections is the necessity of additional surgeries and, in extreme cases, the removal of the implant [17]. A solution to these problems could be the combination of antibiotics with HAp in order to fight the bacteria locally, not allowing them to replicate. However, this is a short-term solution because adsorbed antibiotics are eliminated rather quickly by body fluids [17]. To ensure a long-term antimicrobial effect against post-surgical bacterial infections, the structure of hydroxyapatite must also contain an antimicrobial agent. For this purpose, metal ions such as Ag^+^, Zn^2+^ or Cu^2+^ which are already used in medicine as antimicrobial agents could be integrated into the HAp structure [18,19,20]. Previous studies have demonstrated that silver has high antimicrobial activity, maintaining at the same time low levels of cytotoxicity [17,18,19,20,21,22,23]. Furthermore, there are studies which have proven that silver ions have an oligodynamic effect against Gram-negative and Gram-positive bacteria [17,21,24,25,26].

This research is focused on the study of a new and complex biomaterial that combines the biocompatible properties of hydroxyapatite with the effects of well-known antibiotics such as tetracycline and ciprofloxacin as well as the natural antibiotic effects of silver ions. Tetracycline is a broad-spectrum antibiotic which was discovered more than half a century ago and which is now used for the treatment of many bacterial infections [27]. On the other hand, ciprofloxacin is a second-generation fluoroquinolone used for a number of microbial infections [28]. The study of the structure, morphology and antimicrobial activity of the new biomaterials will help us to better understand their effects against various microbial agents. One of the major goals of our research is to develop a material that could improve the life of patients who have had major orthopedic surgeries, decreasing the risk of post-operatory infections. We hope that combining the traditional approach for treating infections with synthetic antibiotics and the natural approach using natural antibiotics will increase the chance of fighting off bacteria-associated infections.

## 2. Materials and Methods

### 2.1. Tetracycline or Ciprofloxacin Embedded in Silver-Doped Hydroxyapatite Thin Films

The precursors used for the preparation of the thin films based on hydroxyapatite (HAp), silver-doped hydroxyapatite (Ag:HAp) and antibiotics such as tetracycline and ciprofloxacin were calcium nitrate (Ca(NO_3_)_2_·4H_2_O, Sigma Aldrich, Carlsbad, CA, USA), diammonium hydrogen phosphate ((NH_4_)_2_HPO_4_; WakoPure Chemical Industries Ltd., Richmond, VA, USA) and AgNO_3_ (Alpha Aesare, Karlsruhe, Germany, 99.99% purity), as well as tetracycline (C_22_H_24_N_2_O_8_, Sigma Aldrich, Carlsbad, CA, USA, 98% purity) and ciprofloxacin (C_17_H_18_FN_3_O_3_, Alpha Aesare, Karlsruhe, Germany, 98% purity). 

To synthesize pure HAp powder, we used an adapted chemical precipitation method in agreement with previously reported studies [20,26,29]. The resulting solution was stirred vigorously for 72 h at room temperature (RT). Maturation of the final solution based on HAp with or without antibiotics such as tetracycline (T-HAp) or ciprofloxacin (C-HAp) was performed at RT for another 72 h.

The HAp, hydroxyapatite with tetracycline (T-HAp), hydroxyapatite with ciprofloxacin (C-HAp), Ag:HAp, silver-doped hydroxyapatite with tetracycline (T-Ag:HAp) and silver-doped hydroxyapatite with ciprofloxacin (C-Ag:HAp) thin films were prepared using a sol-gel dip coating method on pure Si disk substrates. Controlled amounts of silver nitrate and diammonium hydrogen phosphate were dissolved in ethanol. The solution was stirred vigorously for 24 h at RT. At the same time, a stoichiometric amount of calcium nitrate and tetracycline or ciprofloxacin (previously dissolved in water) was dissolved in ethanol and stirred vigorously for 24 h at RT. The ratio [Ca + Ag]/P was adjusted at 1.67 when the concentration of silver was 𝑥_Ag_ = 0.2 [30,31,32]. The calcium-containing solution was added drop by drop to the phosphate-containing solution. The resulting solution was stirred vigorously for 72 h at RT. Maturation of the final solution based on Ag:HAp with or without antibiotics such as tetracycline (T-Ag:HAp) or ciprofloxacin (C-Ag:HAp) was performed at RT for another 72 h. 

Pure Si disks were cleaned and rinsed in acetone and distilled water. After that, the pure Si disks were dipped in the HAp, T-HAp, C-HAp, Ag:HAp, T-Ag:HAp or C-Ag:HAp and tetracycline (T), ciprofloxacin (C) solution and dried at 40 °C. This operation was repeated three times. Finally, the thin films obtained (HAp, T-HAp, C-HAp, Ag:HAp, T-Ag:HAp, C-Ag:HAp) were dried at 60 °C. The T and C thin films obtained were dried at 40 °C.

### 2.2. Characterization Methods

The structure and morphology of the HAp, T-HAp, C-HAp, Ag:HAp, T-Ag:HAp and C-Ag:HAp thin films were analyzed with a FEI Quanta Inspect F50 scanning electron microscope (FEI, Hillsboro, OR, USA) equipped with an energy dispersive X-ray attachment (EDX). The local qualitative elemental composition of the samples was identified using the Energy Dispersive X-ray analysis. In addition to the conventional SEM image and the local qualitative elemental composition spectra, detailed maps of elemental distribution were also recorded. The crystalline phase structure of the HAp and Ag:HAp thin films were characterized using an X-ray diffractometer Bruker D8 Advance (Bruker, Karlsruhe, Germany). Copper target Cu Kα radiation (wavelength λ = 1.5406 Å) was used. For phase identification, X-ray diffraction (XRD) analysis data were collected in the range of 20° < 2θ < 60° with a step of 0.01° and 34 s measuring time per step. The HAp, T-HAp, C-HAp, Ag:HAp, T-Ag:HAp and C-Ag:HAp thin films were also analyzed by Fourier transform infrared spectroscopy (FTIR) using an SP 100 Perkin Elmer spectrometer (PerkinElmer, Waltham, MA, USA) with a 4 cm^−1^ spectral resolution. The spectra were recorded in Attenuated total reflectance mode (ATR) in the range of 400–2000 cm^−1^ using a Universal Diamond/KRS-5. The glow discharge optical emission spectroscopy (GDOES) technique was used for depth profile analysis, using a GD-Profiler 2™ instrument manufactured by HORIBA Jobin Yvon [33,34,35]. As procedure, using this technique an area of 4 mm of the layer was sputtered by a pulsed RF Ar plasma. The sputtered atoms from the layer were then excited by inelastic collisions in the plasma and the emitted light was monitored in real time providing intensities of the elements present in the investigated sample from the surface down to the substrate as a function of time. The operating conditions were: 650 Pa for pressure and 35 W RF power, working in pulsed mode at 1 kHz pulsing frequency and a duty cycle of 0.25.

### 2.3. Preliminary Observations of Antimicrobial Properties of the HAp, T-HAp, C-HAp, Ag:HAp, T-Ag:HAp and C-Ag:HAp Tetracycline (T) and Ciprofloxacin (C)

To examine the antimicrobial activity of the HAp, T-HAp, C-HAp, Ag:HAp, T-Ag:HAp, C-Ag:HAp, C and T thin films, the diffusion method was used in agreement with Hussain et al. [36]. The *Staphylococcus aureus* (*S. Aureus 0364*) and *Escherichia coli* (*E. coli ATCC 25922*) bacterial strains were grown on nutrient agar medium at a temperature of 37 °C. HAp, T-HAp, C-HAp, Ag:HAp, T-Ag:HAp, C-Ag:HAp, C and T thin films were placed on agar plates with growing bacteria. Incubation was performed for 48 h. After incubation, the inhibition zone was measured. Commercially pure Si substrate (an area of 1 cm^2^) was used as a reference sample.

### 2.4. Antimicrobial Activity of the HAp, T-HAp, C-HAp, Ag:HAp, T-Ag:HAp and C-Ag:HAp, Tetracycline (T) and Ciprofloxacin (C)

The bactericidal properties of the HAp, T-HAp, C-HAp, Ag:HAp, T-Ag:HAp, C-Ag:HAp, C and T thin films were evaluated using *Staphylococcus aureus* (*S. Aureus 0364*) and *Escherichia coli* (*E. coli ATCC 25922*) as models for Gram-positive and Gram-negative bacterial strains. The HAp, T-HAp, C-HAp, Ag:HAp, T-Ag:HAp, C-Ag:HAp, C and T thin films deposited on pure Si disks (1 cm^2^) were exposed to 1.5 mL of bacteria suspension in Phosphate-buffer Saline (PBS). The suspension was collected after 2, 4, 6, 12, 18 and 24 h and incubated on agar medium for 24 h. Finally, the number of CFU/mL (colony forming units per milliliter) was determined.

### 2.5. Statistical Analysis

All the experiments were carried out for the thin film samples in triplicate and repeated three times. The data analysis was carried out using the t-test and analysis of variance (ANOVA). The difference established between samples was appreciated to be significant at *p* < 0.05.

## 3. Results and Discussion

**Figure 1:**
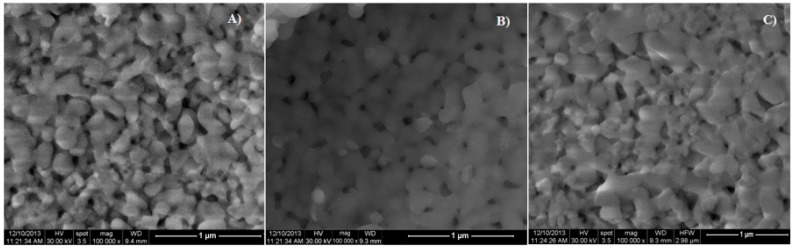
SEM images of HAp (**A**); T-HAp (**B**) and C-HAp (**C**) thin films.

**Figure 2:**
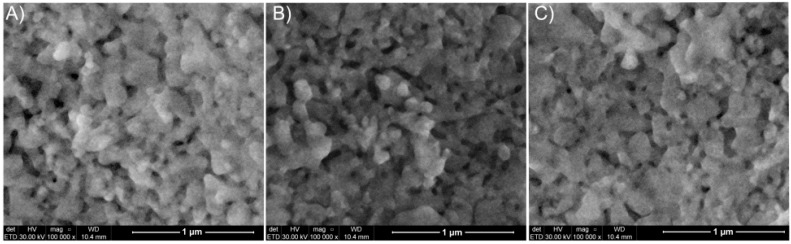
SEM images of Ag:HAp (**A**); T-Ag:HAp (**B**) and C-Ag:HAp (**C**) thin films.

SEM images of HAp, T-HAp, C-HAp, Ag:HAp, T-Ag:HAp and C-Ag:HAp thin films are presented in Figure 1 and Figure 2. The SEM images reveal that the substrate (Si) was covered well by the deposited films. Also, it can be observed that all the samples had a granular morphology with grain dimensions around a hundred nanometers. Moreover, the SEM images showed that the surface morphology was slightly influenced by the presence of the silver, tetracycline and ciprofloxacin.

**Figure 3:**
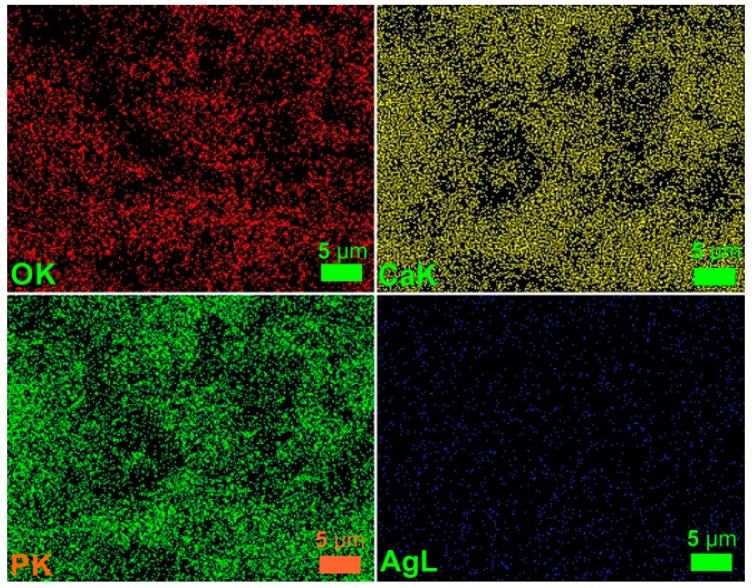
Elemental mapping of Ag:HAP thin film.

By elemental mapping analysis, the silver was evidenced in the Ag:HAp thin film (Figure 3). The homogeneous and continuous composition of the Ag:HAp thin film was also confirmed. Moreover, uniform distribution and good dispersion of Ca, P, O and Ag throughout the surface of the film were observed. 

**Figure 4:**
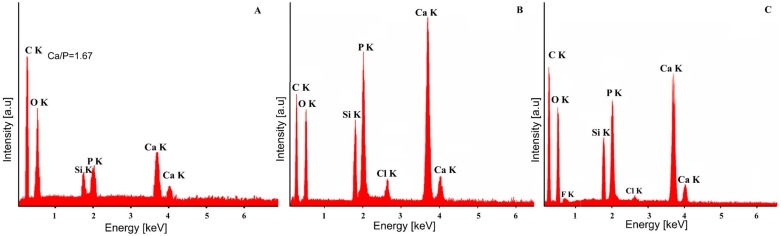
EDX spectra of HAp (**A**); T-HAp (**B**) and C-HAp (**C**) thin films.

**Figure 5:**
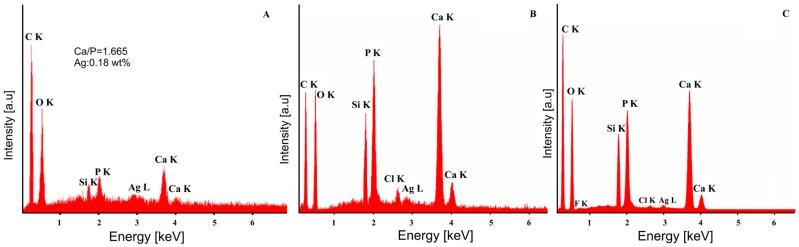
EDX spectra of Ag:HAp (**A**); T-Ag:HAp (**B**) and C-Ag:HAp (**C**) thin films.

The elemental composition of all the samples was investigated using EDX analysis (Figure 4 and Figure 5). The quantitative chemical composition of HAp (Figure 4A) and Ag:HAp (Figure 5A) thin films estimated from EDX analysis was also reported. The EDX spectrum of HAp thin film (Figure 4A) confirms the presence of Ca, O, P and Si. Also, the presence of the constituent elements of the T-HAp thin film (Ca, O, P, N and Si) were evident in the associated EDX spectrum (Figure 4B). Furthermore, Figure 4C shows the EDX spectrum of C-HAp thin film which consists of Ca, O, P, F, N and Si.

Figure 5 exhibits the EDX spectra of Ag:HAp, T-Ag:HAp and C-Ag:HAp composite layers. The EDX spectra presented in Figure 5 reveal that the Ag:HAp, T-Ag:HAp and C-Ag:HAp composite layers are composed of four main elements Ca, P, Ag, and O. The presence of the N constituent element from the tetracycline was observed in T-Ag:HAp thin film (Figure 5B). On the other hand, in the C-Ag:HAp thin films (Figure 5C) the constituent elements from the ciprofloxacin (F and N) were identified as well.

**Figure 6:**
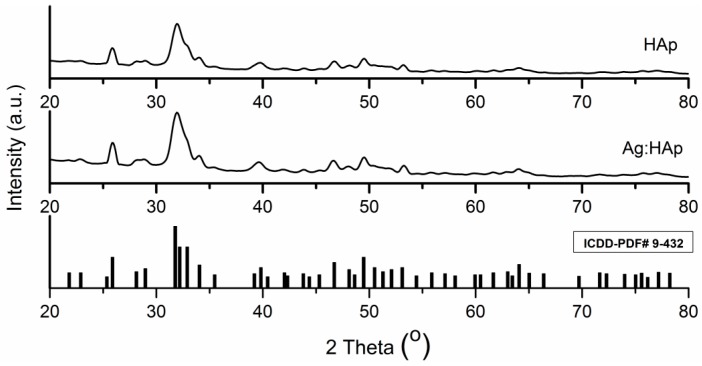
X-ray diffraction patterns of HAp and Ag:HAp thin films.

Figure 6 shows the typical XRD-patterns of HAp and Ag:HAp thin films. The diffraction peaks identified in XRD spectra of HAp and Ag:HAp thin films corresponded to the hexagonal (P63m) lattice of HAp according to the standard JCPDS database (PDF# 09-0432). Furthermore, no silver oxide, other impurities or secondary phases apart from HAp were identified. The diffraction peaks of HAp and Ag:HAp thin films deposited by dipping on pure Si disks did not lead to the formation of other phases.

The EDX spectrum in Figure 5A shows that Ag:HAp thin film was composed mainly of Ca, P, Ag and O. As revealed by the previous XRD analysis, silver and other impurities were not found in the Ag:HAp thin film. All these results demonstrated that silver ions have replaced calcium ions and doped the HAp lattice successfully. Our studies are in good agreement with the previous studies [37] on cerium-doped hydroxyapatite polylactic acid composite coatings on metal substrates.

**Figure 7:**
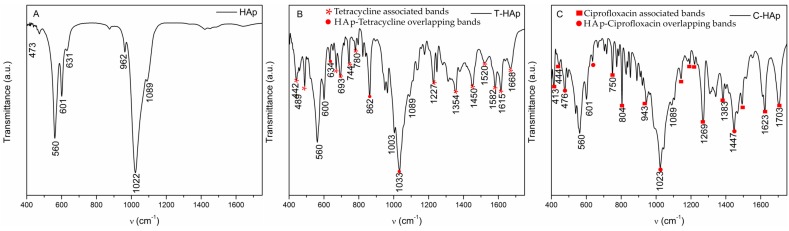
FTIR spectra of HAp (**A**); T-HAp (**B**) and C-HAp (**C**).

**Figure 8:**
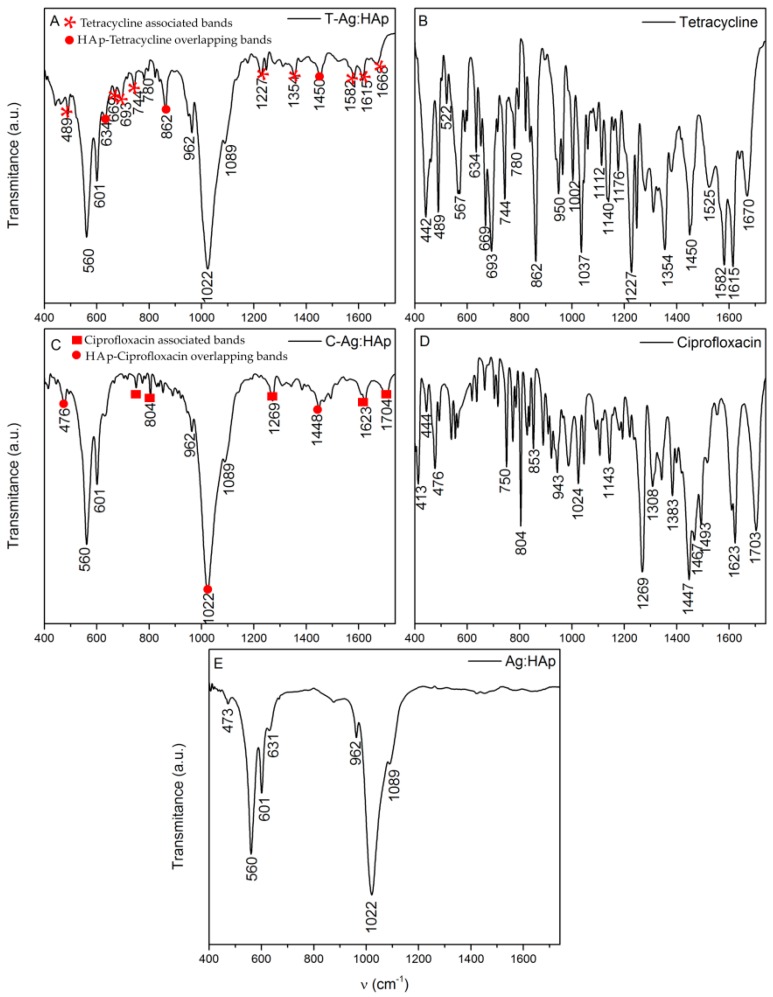
FTIR spectra of Ag:HAp (**E**), T-Ag:HAp (**A**) and C-Ag:HAp (**C**), as well as the spectra of the two antibiotics, Tetracycline (**B**) and Ciprofloxacin (**D**).

In Figure 7 are presented the FTIR spectra of hydroxyapatite (Figure 7A), hydroxyapatite with tetracycline (Figure 7B) and hydroxyapatite with ciprofloxacin (Figure 7C) thin films, while Figure 8 presents the infrared transmission spectra of silver-doped hydroxyapatite (Figure 8D) as well as silver-doped hydroxyapatite with tetracycline (Figure 8A) and ciprofloxacin (Figure 8C) thin films. The spectra of hydroxyapatite (Figure 7A) and silver-doped hydroxyapatite (Figure 8D) highlight the phosphate, carbonate and hydroxyl functional groups present in the prepared sample. All the vibrational bands are characteristic of the pure hydroxyapatite structure. In this context, the main vibrational bands associated with the phosphate PO_4_^3−^ group are found at 472, 561, 602 and 1022 cm^−1^ evidencing the bending modes of the O–P–O bonds [38,39,40,41], while the bands found at 962 cm^−1^ and around 1087 cm^−1^ are characteristic of the non-degenerated symmetric stretching mode of the P–O bond and to the triply degenerated symmetric stretching mode of the P–O bond respectively [38,39,40,41,42]. The carbonate group gives rise to bands in the 870–880 cm^−1^ spectral region [43] and the vibrational mode associated with the presence of the hydroxyl group in the studied sample determines the appearance of the band from 631 cm^−1^ [38].

In the case of the samples which contain hydroxyapatite and antibiotics on one hand and silver-doped hydroxyapatite on the other hand, the registered vibrational bands are associated with both the apatite structure and the corresponding antibiotic structure. It can easily be observed that the two spectra of the Ag:HAp (*x*_Ag_ = 0.2) with tetracycline (Figure 8A) and ciprofloxacin (Figure 8C) thin films are very similar to the spectrum of Ag:HAp (*x*_Ag_ = 0.2) thin film (Figure 8D). In contrast to the case of silver-doped hydroxyapatite (Figure 8A,C), in the case of hydroxyapatite (Figure 7B,C) the bands specific to the structures of tetracycline and ciprofloxacin appear to be more intense, which suggests that the incorporation of silver ions in the apatitic structure has a strong impact on the process of embedding antibiotics. Almost all the vibrational bands described earlier, characteristic of the apatitic structure, can be found. However, in the case of the thin films containing hydroxyapatite and silver-doped hydroxyapatite with tetracycline (Figure 7B and Figure 8A), the band from 472 cm^−1^ has disappeared, while in the case of the thin films containing hydroxyapatite and silver-doped hydroxyapatite with ciprofloxacin (Figure 7C and Figure 8C), this band is found to be shifted, at around 476 cm^−1^ and the band from 862 cm^−1^ is missing. Meanwhile, the additional bands observed in the four spectra are associated with functional groups characteristic of the two antibiotics. With their quite complicated chemical structures, both types of antibiotics present many types of vibrational modes.

Therefore, in the case of the hydroxyapatite and silver-doped hydroxyapatite with tetracycline thin films (Figure 7B and Figure 8A), most of the additional bands observed in the spectra correspond to the aromatic ring stretching vibrations (1452, 1584, 1615, 1668 cm^−1^) [44]. The band found at around 1358 cm^−1^ corresponds to either C–O stretching, symmetric CH_3_ bending mode, or to the terminal dimethyl bending vibrational mode [44], while the band from around 1234 cm^−1^ corresponds to the C–N stretching mode or to the C–C stretching mode [44]. Also, the influence of tetracycline is shown by the presence of the bands between 650 and 900 cm^−1^ which are associated with the aromatic =C–H deformations [45]. Therefore, the bands from 489, 669 and 693 cm^−1^ correspond to the out-of-plane aromatic ring deformation, while the band from 634 cm^−1^ shows the in-plane ring deformation [45].

In the case of the hydroxyapatite and silver-doped hydroxyapatite with ciprofloxacin thin films (Figure 7C and Figure 8C), the additional bands observed in the infrared spectra, except for the ones associated with hydroxyaptite and silver-doped hydroxyapatite (Figure 8E), are characteristic of the antibiotic chemical structure. Therefore, in the 1000–1050 cm^−1^ spectral range bands can be found that are characteristic of the stretching vibrations of the C–F bond from the fluorine group, between 1250 and 1300 cm^−1^ are present the bands of the O–H bending vibrations of the hydroxyl group, while in the 1400–1450 cm^−1^ range the bands of the carbonyl group C–O stretching vibration can be found and between 1600 and 1650 cm^−1^ the N–H bending vibrations of the quinolines are present [46]. Also, in the spectral region between 1700 and 1750 cm^−1^, the vibrational bands are associated with the C=O stretching of the CO group of acids [46]. Some of the bands associated with the ciprofloxacin structure from the C-HAp and C-Ag:HAp thin films (Figure 7C and Figure 8C) appear slightly shifted compared to the ciprofloxacin spectrum (Figure 8D). This behavior might be attributed to the apatitic structure of hydroxyapatite and silver-doped hydroxyapatite, respectively.

Considering the complexity of the chemical structures of the two antibiotics studied, it is sometimes hard to distinguish the vibrational bands associated with each functional group since bands can be found in the same spectral region that are characteristic of different chemical bond vibrations.

It can easily be observed that the addition of the two antibiotics has influenced the structure of the silver-doped hydroxyapatite, creating new vibrational bands characteristic of the specific chemical composition. These additional bands are much more attenuated than those observed in the spectra of the two antibiotics, but their presence suggests a good interaction with the apatitic structure.

The glow discharge optical emission spectroscopy (GDOES) technique was used for depth profile analysis of the HAp, T-HAp and C-HAp, Ag:HAp, T-Ag:HAp and C-Ag:HAp composite layers deposited on silicon substrates. Through these measurements the chemical elements contained in the coating and their distribution throughout the film could be identified. As the sputtering rate is material-dependent and varies during the elemental depth profiling measurement, an accurate conversion of sputtering time into sputtered depth is not straightforward [47,48]. 

**Figure 9:**
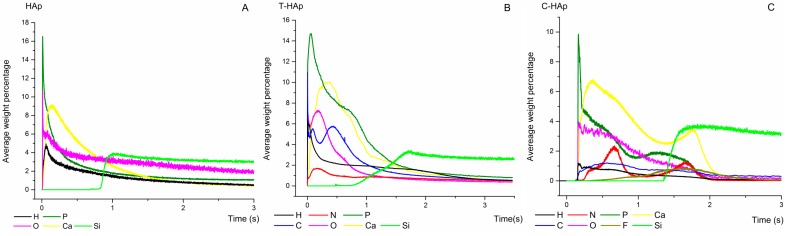
Depth profile curves of HAp (**A**); T-HAp (**B**) and C-HAp (**C**) thin films.

**Figure 10:**
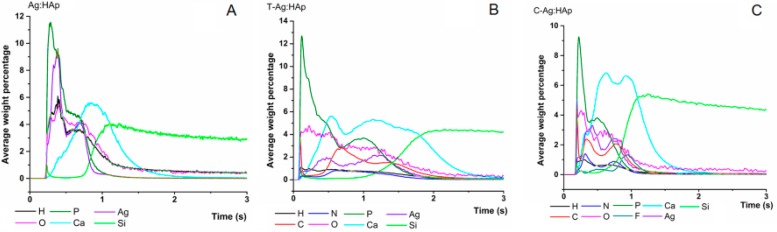
Depth profile curves of Ag:HAp (**A**); T-Ag:HAp (**B**) and C-Ag:HAp (**C**) thin films.

Figure 9 and Figure 10 present the depth profile curves of HAp, T-HAp and C-HAp with their respective Ag:HAp, T-Ag:HAp and C-Ag:HAp coatings.

The Ca, P, O, Ag and H depth profile curves were presented as they were identified in the HAp respectively Ag:HAp thin films from Figure 9A and Figure 10A. The HAp/Si and Ag:HAp/Si substrate interfaces were marked by an increase of the intensity of Si depth profile curve and a decrease of the intensities of all the other chemical elements.

In Figure 9B,C, and Figure 10B,C it could be observed that the depth profile curves of the elements identified in the T-HAp, C-HAp, T-Ag:HAp and C-Ag:HAp composite layers had similar behavior. There was no sharp delimitation between the Ca, Ag, P, O and H depth profile curves, specific to HAp and Ag:HAp, and the depth profile curves of C, N, O, F and H atoms, associated with the structures of the two antibiotics. Usually, such results could indicate either the roughness of the interface (as the GD averages the signals over the entire erosion zone), the flatness of the crater bottom or the formation of a composite material. For the present analysis, the GDOES operating conditions mentioned above provide a flat crater bottom, while the interface did not indicate any roughness.

The Ca depth profiles observed in Figure 9B,C and Figure 10B,C are broader than those presented in Figure 9A and Figure 10A. This could be an indication of Ca atom redistribution in the bulk of the thin layers that contain antibiotics. The simultaneous increase of the Si profile with the decrease of all the other element profiles marks the coating/substrate interface [47,48]. 

Correlating these observations, we assume that during the deposition process of T-HAp, C-HAp, T-Ag:HAp and C-Ag:HAp layers, the incorporation of the antibiotics into the HAp and Ag:HAp structure took place, thus forming a composite coating.

The IR spectral analysis presented above showed that the IR bands characteristic of tetracycline and ciprofloxacin structures identified in the spectra acquired for thin films based on hydroxyapatite and silver-doped hydroxyapatite with antibiotics (Figure 8) are slightly shifted compared to those present in the antibiotics IR spectra (Figure 8). An explanation for this behavior could be the appearance of some chemical changes into the apatitic structure of the pure hydroxyapatite and silver-doped hydroxyapatite. The distribution of Ca, P, O, Ag, C, N, F, H elements present in the investigated layers indicated by their elemental depth profiles from Figure 9 and Figure 10 confirms the IR spectral analysis regarding the incorporation of antibiotics into the HAp and Ag:HAp structure.

**Figure 11:**
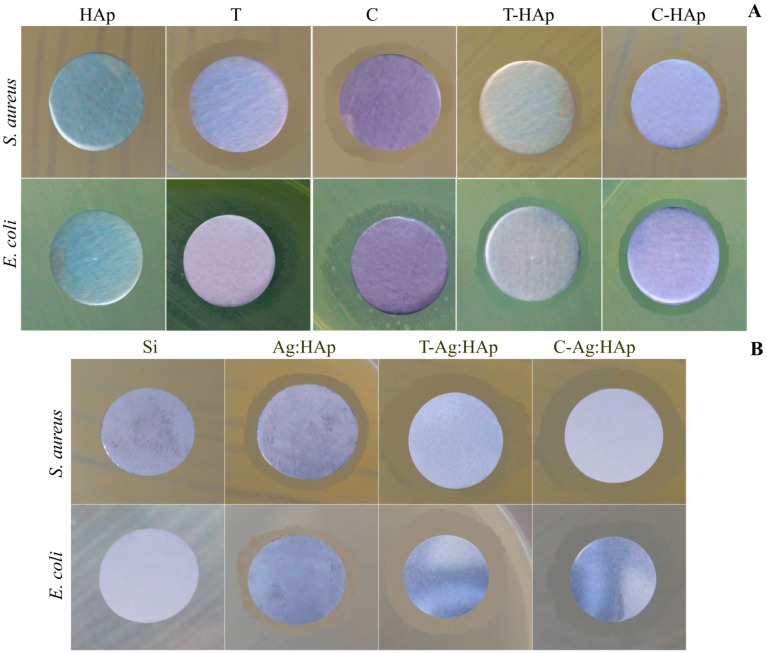
Antibacterial activity against *S. aureus* *0364* and *E. coli ATCC 25922* cultures of: (**A**) HAp, T-HAp and C-HAp, Ciprofloxacin, Tetracycline thin films and (**B**) Ag:HAp, T-Ag:HAp, C-Ag:HAp thin films.

The efficacy of HAp, T-HAp, C-HAp, Ag:HAp, T-Ag:HAp, C-Ag:HAp, C and T thin films was evaluated against Gram-positive and Gram-negative bacterial strains such as *S. aureus 0364* and *E. coli ATCC 25922* (Figure 11).

The pure HAp thin films (Figure 11A) did not show any antimicrobial activity after 48 h of incubation in presence of *E. coli ATCC 25922* and *S. aureus 0364* cultures. By contrast, after 48 h of incubation in presence of *E. coli ATCC 25922* and *S. aureus 0364* cultures, the T-HAp and C-HAp thin films samples showed inhibition zones (Figure 11A). The images of the *E. coli ATCC 25922* and *S. aureus 0364* cultures after being exposed to the Ag:HAp, T-Ag:HAp and C-Ag:HAp thin films for 48 h are also presented in Figure 11B. It can be seen that the migration of the Ag^+^ ions to the organic medium after 48 h of incubation occurs differently, depending on the bacterial strain and the film composition. Due to the valence difference between Ca and Ag ions in the anionic site, a vacancy will be created. One of the Ca^2+^ sites can be substituted by Ag^+^ in the presence of silver [49]. The explanation for this phenomenon would be that the *r*_ion_ (Ag^+^) is greater than *r*_ion_ (Ca^2+^). Moreover, different zones of inhibition were observed in the case of *E. coli ATCC 25922* and *S. aureus 0364* cultures after 48 h of exposure to the Ciprofloxacin and Tetracycline thin films (Figure 11A).

After 48 h of incubation, for the *E. coli ATCC 25922* culture, the Ag:HAp, T-Ag:HAp and C-Ag:HAp thin films presented different zones of inhibition (Table 1). After 48 h incubation for the *S. aureus 0364* culture it can be seen that the mean zone of inhibition of Ag:HAp, T-Ag:HAp and C-Ag:HAp thin films increased from Ag:HAp to T-Ag:HAp (Table 1). The analysis of the antimicrobial activity of Ag:HAp, T-Ag:HAp and C-Ag:HAp thin films against *E. coli ATCC 25922* and *S. aureus 0364* bacterial strains regarding the mean zone of inhibition was presented in Table 1.

**Figure 12:**
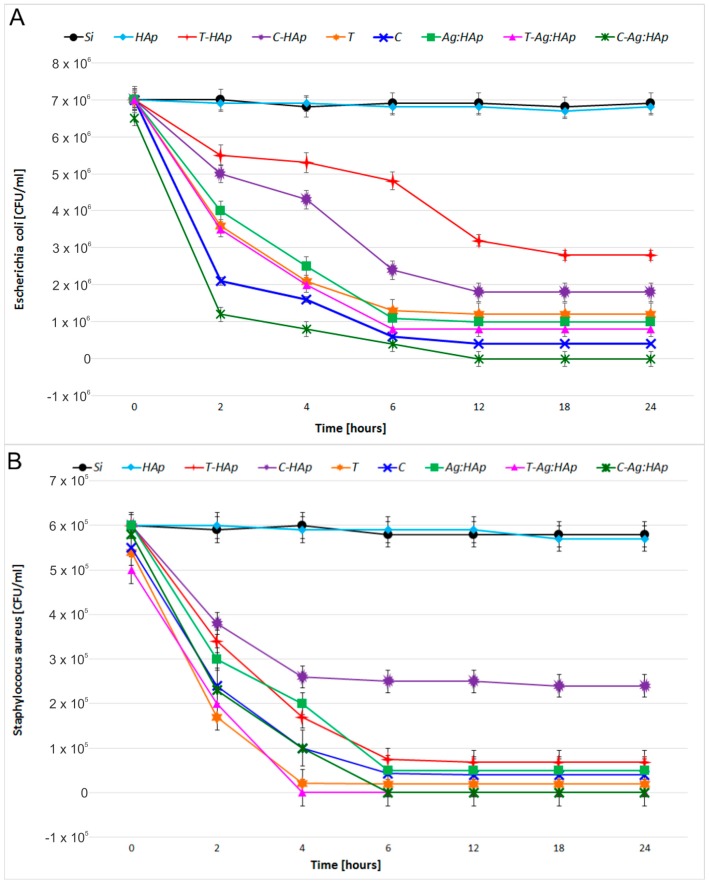
Bactericidal effects of time of contact with the surface of HAp, T-HAp, C-HAp, Ag:HAp, T-Ag:HAp, C-Ag:HAp, C and T thin films for *E. coli ATCC 25922* (**A**) and *S. aureus* *0364* (**B**).

For the *E. coli ATCC 25922* and *S. aureus 0364* bacterial strains, the biological activity of HAp, T-HAp, C-HAp, Ag:HAp, T-Ag:HAp, C-Ag:HAp, C and T thin films was evaluated depending on the time of contact. The *E. coli ATCC 25922* and *S. aureus 0364* bacterial strain were used to study the in vitro antimicrobial properties of HAp, T-HAp and C-HAp (Figure 12). The bactericidal effects of the time of contact with the surfaces of Ag:HAp, T-Ag:HAp, C-Ag:HAp, Tetracycline (T) and Ciprofloxacin (C) thin films for *E. coli ATCC 25922* and *S. aureus 0364* were also presented in Figure 12.

As it can be seen in Figure 12, when the antimicrobial effect of HAp thin film against *E. coli ATCC 25922* and *S. aureus 0364* was tested, the HAp thin film did not present any antibacterial activity in the range of 0–24 h. It can be said that the HAp layer did not show antibacterial properties. These results are in good agreement with the previous studies presented by Kim et al. [29,50]. Therefore, according to Zeng et al. [51] the pure HAp layer presents several disadvantages, because it had no antimicrobial activity, which affects its long term stability and engenders implant failures. Hence, the success of an orthopedic implantable biomaterial depends not only on the bone implant integration but also on the presence of a sterile environment around the implant, which will prevent implant related bacterial infections. Consequently, we tried to create a layer with antimicrobial properties, thus improving the antibacterial properties of HAp by doping it with antibiotics (ciprofloxacin and tetracycline) and by substitution of Ca^2+^ ion with silver ions, which have been found to effectively inhibit the postoperative infections.

The T-HAp and C-HAp thin films samples showed an antibacterial effect against *E. coli ATCC 25922* and *S. aureus 0364* (Figure 12). The presence of ciprofloxacin in C-HAp thin films and tetracycline in T-HAp thin films led to an increase in antibacterial effect against both types of bacteria.

A drastic reduction of the CFU number of the *E. coli ATCC 25922* cultures was obseved after 6 and 12 h of exposure to T-HAp and C-HAp thin films (Figure 12A). The C-HAp thin film exhibited a good bacteriostatic effect against the tested bacterial strain *E. coli ATCC 25922* after 18 h (Figure 12A). Furthermore, a strong decrease of the CFU number of the *S. aureus 0364* cultures was noticed after 6 h of exposure to T-HAp and C-HAp thin films (Figure 12B). Overall, a strong bacteriostatic effect of T-HAp thin films against *S. aureus 0364* was observed (Figure 12B).

Ag:HAp, T-Ag:HAp and C-Ag:HAp thin films have presented a rapid antimicrobial activity against *E. coli ATCC 25922* which led to a drastic reduction in the number of cells after 6 h (Figure 12A). Nevertheless, the bactericidal effect of Ag:HAp and T-Ag:HAp against *E. coli ATCC 25922* was incomplete. Considering the fact that almost 12% of the *E. coli* bacterial strains have become resistant to various antibiotics, among them being tetracycline [52], the T-Ag:HAp thin film exhibited a good bacteriostatic effect against the tested bacterial strain *E. coli ATCC 25922*. After 6 h of exposure, the CFU number decreased significantly. One explanation for this behavior could be the combination of silver ions and tetracycline. On the other hand, ciprofloxacin is a well-known antibiotic used frequently for the treatment of infections associated with the presence of *E. coli* bacterial strains. Therefore, the bacteriological eradication of the *E. coli ATCC 25922* was achieved after 12 h of exposure to the C-Ag:HAp thin film.

In contrast to the effect on *E. coli ATCC 25922*, the Ag:HAp, T-Ag:HAp and C-Ag:HAp thin films rapidly killed *S. aureus 0364* cells and the bactericidal effect of T-Ag:HAp and C-Ag:HAp was complete after 4 and 6 h, respectively (Figure 12B). Unfortunately, in this case, the bactericidal effect of Ag:HAp against *S. aureus 0364* was incomplete (Figure 12B). Comparing the influence of both Ag:HAp and T-Ag:HAp thin films against *S. aureus 0364*, it can be concluded that the presence of tetracycline led to an increase of the bactericidal effect. It can also be seen that presence of ciprofloxacin in C-Ag:HAp thin films increases the bactericidal effect against *S. aureus 0364* in comparison to the effect of Ag:HAp thin film. All the same, in this case, the bactericidal effect was completed after 6 h, two hours later than in the case of T-Ag:HAp thin film.

Tetracycline and ciprofloxacin thin films showed a significant increase in antimicrobial effect against *E. coli ATCC 25922* and *S. aureus 0364*. The antimicrobial effects of tetracycline thin films increased after 6 h and the CFU number decreased significantly. However, the number of CFU observed for tetracycline thin films was slightly higher compared to the number of CFU observed in the case of Ag:HAp, T-Ag:HAp and C-Ag:HAp thin films (Figure 7A). The bacteriostatic effect of ciprofloxacin thin films against *E. coli ATCC 25922* was observed after 12 h. Anyway, the bactericidal effect was not observed. The bactericidal effect against *E. coli ATCC 25922* was observed only for the C-Ag:HAp thin film. Tetracycline and ciprofloxacin thin films rapidly killed *S. aureus 0364* cells and the bactericidal effect of tetracycline was complete after 6 h (Figure 12B). Moreover, the bacteriostatic effect of ciprofloxacin against *S. aureus 0364* was noticed (Figure 12B). On the other hand, the bactericidal effect of T-Ag:HAp and C-Ag:HAp was also observed against *S. aureus 0364* cells after 6 h (Figure 12B).

HAp has been studied as a bone substitute without needing to be removed afterwards [53]. However, the use of pure HAp in various implants has major disadvantages due to lack antimicrobial activity, which affects long-term stability and might lead to failures. According to previous studies [53,54,55] the lack of the antimicrobial activity leads to bacterial adhesion to the biomaterial surfaces, reduces the success rate of surgery and sometimes increases patient's mortality/prognosis. Recently, researchers have tried to dope HAp with various antibiotics or antibacterial agents in order to facilitate its use in bone substitutions and in the case of infected bone defects. On the other hand, it was observed that more and more Gram-positive and Gram-negative bacterial strains such as *Staphylococcus aureus* and *Escherichia coli* have developed resistance to a wide range of antibiotics used in numerous antimicrobial treatments. As a result, the presence of a sterile environment around the implant plays an important role. The success of an implantable biomaterial depends on both bone implant integration and the presence of a sterile environment around the implant to prevent bacterial infections. As a result, in recent years scientists tried improving the antimicrobial properties of HAp by substituting Ca^2+^ ions with different ions like Ag^+^ [20], Zn^2+^ [56] and Ce^3+^ [19]. The synthesis of hydroxyapatite doped with different elements such as silver, zinc, cerium, samarium, etc. [19,20,56,57], can lead to new antimicrobial materials being obtained that could be used to produce coatings able to inhibit postoperative infections. Therefore, it is desirable for the implantable biomaterials to have antimicrobial properties in combination with good osseointegration property. It is well known that the silver ions, Ag^+^, have been used in different biomedical fields for a long time due to their antimicrobial properties. As well, it is known that silver-based compounds may have a bactericidal effect on several species of bacteria that cause serious disease [58]. More than that, previous studies [59,60] have shown that the presence of silver ions in the HAp matrix increases the mechanical properties. Feng et al. [61] reported the antibacterial studies on Ag:HAp samples obtained by soaking HAp samples in AgNO_3_ solutions. When this process was performed, the Ca^2+^ ions were substituted by Ag^+^ ions. Moreover, precedent studies reported that the final Ag:HAp samples showed antibacterial effects against Gram-positive and Gram-negative bacterial strains such as *Staphylococcus aureus* and *Escherichia coli*. The silver-doped HAp in the form of implantable biomaterial could be used to hinder bacterial adherence on biomaterial surface conducting to a good tissue regeneration.

Consequently, in the last few years, attempts have been made in order to create new materials that can simplify the treatment of infections. Thin layers based on HAp doped with silver and antibiotics could hamper the growth of antibiotic resistant bacterial. At the same time, this new material based on silver-doped HAp could lead to an increase of the bactericidal effect. The substantial reduction of the number of CFU and the increase of the bactericidal effect for the Ag:HAp, T-Ag:HAp and C-Ag:HAp after exposure to *E. coli ATCC 25922* and *S. aureus 0364* confirmed the importance of our research.

## 4. Conclusions

The goal of this research was to study the structure, morphology and the antibacterial effect of different thin films based on hydroxyapatite, silver-doped hydroxyapatite and tetracycline/ciprofloxacin embedded in hydroxyapatite and silver-doped hydroxyapatite, respectively. By incorporation of these antibiotics, the bactericidal effect of the new materials against *E. coli ATCC 25922* and *S. aureus 0364* bacterial strains was induced. Therefore, these materials could be good candidates for future use as local drug delivery systems without changing their biocompatibility. 

The morphological studies represented by SEM micrographs suggested that all the studied samples have granular shapes, since the surface of the T-HAp, C-HAp, T-Ag:HAp and C-Ag:HAp samples are influenced by the presence of the antibiotics. Moreover, the elemental mapping showed a uniform distribution of all the elements contained in the studied sample throughout the surface of the film. The elemental composition of HAp, Ag:HAp, T-HAp, T-Ag:HAp, C-HAp and C-Ag:HAp was also investigated by EDX measurements. The results suggested that all the elements found are associated with the structures of hydroxyapatite and silver-doped hydroxyapatite combined with the two antibiotics used. Structural characterization of the HAp and Ag:HAp thin films has been performed by XRD analysis. The obtained data revealed that the structures of these thin films correspond to the hexagonal (P63m) lattice of pure hydroxyapatite. The diffraction spectra suggested that no other secondary phases or impurities were detected. Furthermore, the functional groups present in each sample were identified using FTIR spectroscopy. The main vibrational bands found in the spectra registered for the six thin films (HAp, Ag:HAp, T-HAp, T-Ag:HAp, C-HAp and C-Ag:HAp) have been associated with the apatitic structure, with small alterations in the four spectra of the samples containing antibiotics. The presence of the antibiotics has led to the appearance of various new vibrational bands characteristic of their specific chemical composition. These new bands suggest the incorporation of antibiotics into the apatitic structure. Depth profile analysis of the HAp, Ag:HAp, T-HAp, T-Ag:HAp, C-HAp and C-Ag:HAp composite layers deposited on silicon substrates have been performed using the GDOES technique. The results suggested that during the deposition process of T-HAp, T-Ag:HAp, C-HAp and C-Ag:HAp layers, the two antibiotics have been successfully incorporated into the HAp and Ag:HAp structures, thus forming a composite coating.

The antimicrobial efficacy of HAp, Ag:HAp, T-HAp, T-Ag:HAp, C-HAp and C-Ag:HAp thin films was evaluated against *S. aureus 0364* and *E. coli ATCC 25922* bacterial strains. Also, the bactericidal effect of the prepared thin films was evaluated, depending on the time of exposure to the bacterial strains. It was found that the newly created materials based on HAp and Ag:HAp and antibiotics provided a strong bactericidal effect against *S. aureus 0364* and a reduction of the CFU number after exposure to *E. coli ATCC 25922* which was caused by the bacteriostatic effect. In the case of *E. coli ATCC 25922*, the T-Ag:HAp thin film reduced drastically the CFU number after 6 h of exposure. On the other hand, the bacteriological eradication was achieved after the C-Ag:HAp thin film was exposed for 12 h to *E. coli ATCC 2592*2. In the case of *S. aureus 0364*, the bactericidal effect of T-Ag:HAp and C-Ag:HAp thin films was even more pronounced. The exposure of C-Ag:HAp thin film to *S. aureus 0364* for 6 h led to a complete annihilation of the bacterial strain, while in the case of T-Ag:HAp thin film, an exposure of only 4 h was necessary to achieve complete annihilation. In the case of HAp, the presence of ciprofloxacin increased the antibacterial effect against against both types of bacteria, while the presence of tetracycline induced a strong bacteriostatic effect against *S. aureus 0364.*

Our results suggest that combining hydroxyapatite and silver-doped hydroxyapatite with some of the most popular antibiotics, used for a number of bacterial infections worldwide, could produce powerful composites capable of eradicating various bacterial strains. Also, the antimicrobial activity of both antibiotics was boosted by the presence of silver ions in the composition of the studied thin films. Our research has established the premises for a new material that could be used in the future for various biomedical applications.

## Figures and Tables

**Table 1:** Antibacterial activity of HAp, T-HAp, C-HAp, Ag:HAp, T-Ag:HAp, C-Ag:HAp, Ciprofloxacin (C) and Tetracycline (T) thin films against *E. coli ATCC 25922* and *S. aureus 0364* bacterial strains.

Bacterial Strains	Thin Films Samples	Mean Zone of Inhibition (mm)
*E. coli ATCC 25922*	Si (control)	0
	HAp	0
	T-HAp	17 ± 0.3
	C-HAp	19 ± 0.5
	Ag:HAp	23 ± 0.6
	T-Ag:HAp	25 ± 0.7
	C-Ag:HAp	28 ± 0.2
	T	20 ± 0.4
	C	26 ± 0.3
*S. aureus 0364*	Si (control)	0
	HAp	0
	T-HAp	26 ± 0.5
	C-HAp	24 ± 0.5
	Ag:HAp	30 ± 0.6
	T-Ag:HAp	40 ± 0.7
	C-Ag:HAp	35 ± 0.4
	T	33 ± 0.5
	C	31 ± 0.7
